# In Vitro and In Vivo Biocompatibility Studies of a Cast and Coated Titanium Alloy

**DOI:** 10.3390/molecules25153399

**Published:** 2020-07-27

**Authors:** Ursula Sommer, Stephan Laurich, Lucie de Azevedo, Katharina Viehoff, Sabine Wenisch, Ulrich Thormann, Volker Alt, Christian Heiss, Reinhard Schnettler

**Affiliations:** 1 Experimental Trauma Surgery, Justus-Liebig-University Giessen, Aulweg 128 (ForMED), 35392 Giessen, Germany; st.laurich@asklepios.com (S.L.); luciedeazevedo@gmx.de (L.d.A.); katharina.viehoff@e.mail.de (K.V.); ulrich.thormann@chiru.med.uni-giessen.de (U.T.); christian.heiss@chiru.med.uni-giessen.de (C.H.); 2 Clinic of Small Animals, c/o Institute of Veterinary Anatomy, Histology and Embryology, Justus-Liebig-University Giessen, Frankfurter Strasse 98, 35392 Giessen, Germany; sabine.wenisch@vetmed.uni-giessen.de; 3 Department of Trauma, Hand and Reconstructive Surgery, University Hospital Giessen-Marburg GmbH, Campus Giessen, Rudolf-Buchheim-Str. 7, 35385 Giessen, Germany; 4 Department of Trauma Surgery, University Medical Center Regensburg, Franz-Josef-Strauss-Allee 11, 93053 Regensburg, Germany; volker.alt@ukr.de; 5 Department of Oral and Maxillofacial Surgery, Division for Regenerative Orofacial Medicine, University Hospital Hamburg-Eppendorf, 20246 Hamburg, Germany; reinhard.schnettler@uke.de

**Keywords:** bone, implant, Ti-6Al-7Nb, rat, sheep, cell culture, biocompatibility

## Abstract

The biocompatibility of a cast porous and with a calcium titanate reaction layer functionalized titanium alloy (Ti-6Al-7Nb) was tested by means of cell culture, and a small (rat) and large animal (sheep) model. The uncoated titanium material served as a control. In-vitro tests included the validation of osteoblast-like cells attached to the surface of the material with scanning electron microscopy and immunofluorescence of cytoskeletal actin as well as their osteogenic development, the ability to mineralize, and their vitality. Following the in-vitro tests a small animal (rat) and big animal (sheep) model were accomplished by inserting a cylindrical titanium implant into a drill hole defect in the femoral condyle. After 7, 14, and 30 days (rat) and 6 months (sheep) the condyles were studied regarding histological and histomorphometrical characteristics. Uncoated and coated material showed a good biocompatibility both in cell culture and animal models. While the defect area in the rat is well consolidated after 30 days, the sheep show only little bone inside the implant after 6 months, possibly due to stress shielding. None of the executed methods indicated a statistically significant difference between coated and uncoated material.

## 1. Introduction

The ability to regenerate is the outstanding property of the bone tissue in the difference to all other tissues [1]. The regenerative reconstitution of bones does not occur, however, if a defect exceeds a critical size or the distance between the ends of a fracture is too large. Approximately 10% of all fractures requires restoration interventions [2]. The healing processes of iatrogenic bone defects, which can occur after tumor or cyst resection, also require a surgical treatment by filling in the defects. This also applies to bony defects, which arise in the course of post-traumatic healing complications and infections [3]. Ideally, the filling material should be stable immediately after implantation, moreover, biocompatible, and absorbable. So far, only autologous bone met these requirements and is still considered the gold standard for defect filling. Nevertheless, the use of autologous bone has disadvantages. The harvesting requires a second surgical intervention, which always poses a risk to the patient. In addition, pain [4], bruising, subsequent bleeding, wound healing disorders, and infections can occur at the harvesting site [5]. Furthermore, only a limited amount of a person’s own bone is available. All these disadvantages limit the possibility to treat bone defects with the body’s own material. Alternatively, allogenic bone grafts can be used. However, they carry the risk to trigger immunological reactions in the recipient. Also, the transmission of infectious material such as hepatitis viruses or HIV can never be safely excluded. Last but not least, the procurement, sterilization, and storage of allogenic bones require an extremely expensive infrastructure [3,6]. Within the large group of absorbable and nonabsorbable bone substitutes, calcium phosphate-based materials show excellent biocompatibility. Their advantage is the osteoconductive promotion of the healing process [3,7,8], whereas the disadvantage, even of the sintered hydroxyapatite ceramics, is a high brittleness and a lack of mechanical stability [9,10,11]. This significantly limits the clinical use of such materials. In particular, mechanically heavily loaded skeletal sections, such as the spine, the calcaneus, and the long bones of the extremities require the use of more stable implants. Titanium and titanium alloys represent metallic biomaterials with excellent mechanical properties and good biocompatibility [12,13,14]. The surface of the metallic material influences the extent of its biocompatibility. On one hand, hydroxyl groups associated with titanium influence the formation of a hydroxyapatite layer on its surface [15]. On the other hand, the natural formation of a superficial oxide layer promotes cellular activities in vitro and the osseointegration of the titanium implant in host bone in vivo [16,17,18,19,20,21]. Ultimately, these improved biological properties are based on an increase in surface roughness and the implementation of calcium and phosphate in the surface layer. A rough surface improves the cell adhesion and the mechanical anchoring of the implant in the tissue, while the calcium and phosphate sources stimulate the synthesis of osteoblasts and thus support osseointegration [14]. In this way, the formation of hydroxyl carbonate crystal agglomerates on titanium surfaces also contributes to an intensive bond between the implant and the bone [21]. Hydroxyapatite (HA) and calcium phosphate coatings on titanium implants increase the biocompatibility and osteoconductivity [22,23,24,25,26,27,28] due to the crystallographic similarity of HA to inorganic bone matrix [29,30]. A calcium coating should improve the biocompatibility and osseointegration of a metallic implant. In contrast to uncoated titanium, Ca ions, which were associated with the titanium surface by ion implantation, promote the formation of a calcium phosphate layer along the surface of the material in the presence of fibroblasts from MC3T3 line [31,32]. Additionally, there is an accelerated formation of osteoid in the presence of calcium titanate [31]. Ca ions also stimulate cells of the osteoblast line to express Bone Morphogenetic Protein (BMP)-2 and -4 and to synthesize collagen I [33,34].

To evaluate the possible benefit of a calcium titanate reaction layer in a critical size defect in bone, we chose the rat as a small animal model, and the sheep as a large animal model. Before starting the animal models, a cell culture test was carried out to determine the biocompatibility of the cast and functionalized titanium alloy material. In all investigations, uncoated specimens served as a control in order to rule out possible effects of the selected alloy or the casting process on the assessment of the biocompatibility of the calcium titanate layer.

## 2. Results

### 2.1. Cell Culture

#### 2.1.1. Cell Adherence

**Figure 1:**
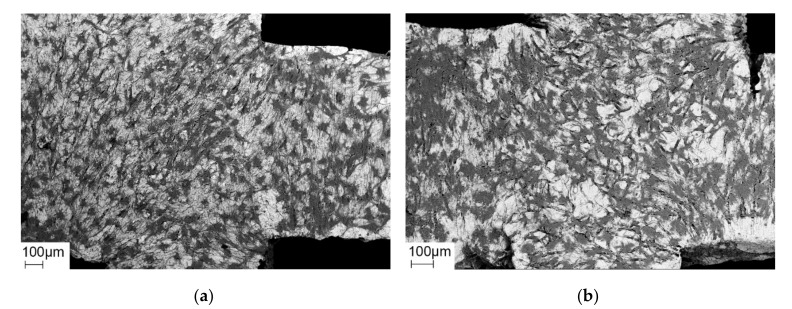
Scanning electron microscopic (SEM): dark cells are scattered all over the bright surface of the coated (**a**) and uncoated (**b**) grid.

**Figure 2:**
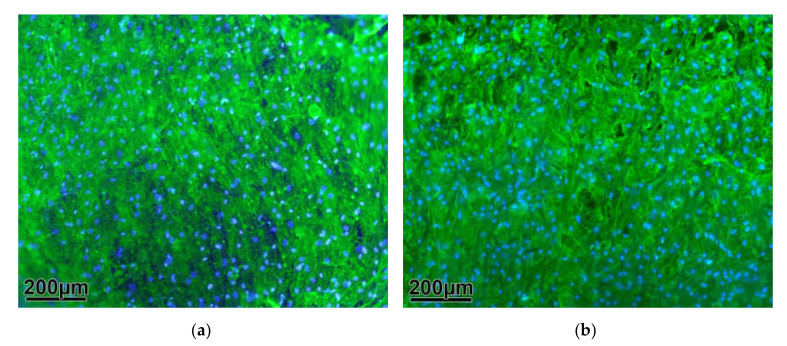
Immunofluorescence microscopic (IF): numerous cells attached to the coated (**a**) and uncoated (**b**) grid surface are visible due to actin immunofluorescence (green).

The osteogenically differentiated cells are able to adhere to the metal surface and build a dense cell carpet, regardless of whether the titanium alloy is coated or uncoated, as can be seen in scanning electron microscopic (SEM, Figure 1) and immunofluorescence microscopic (IF, Figure 2) examinations.

#### 2.1.2. Proliferative Capability

**Figure 3:**
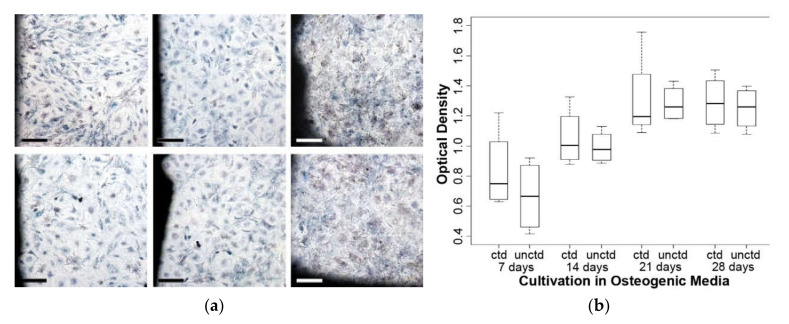
MTT: The pictures (**a**) were taken before measuring optical density to illustrate the distribution of the cells and the position in close proximity to the metal on the left side. Top row: coated, 7, 14, and 28 days. Bottom row: uncoated, 7, 14, and 28 days; bar = 200 µm (**b**) There is no significant difference between the mitochondrial activity of the cells in the presence of the coated (ctd) and uncoated (unctd) material.

With the help of the MTT (3-(4,5-dimethylthiazol-2-yl)-2,5-diphenyltetrazolium bromide) test, differences between the proliferative capabilities of the cells in the presence of the coated and uncoated metals become visible, because the cell’s own dehydrogenases reduce the tetrazolium salt used in this experiment into an insoluble blue colored Formazan (Figure 3a). During the conversion there is a linear relation between the resulting optical density of the blue dye and the mitochondrial activity. Although the strength of the optical density increases noticeably from 7 to 21 and weaker from 21 to 28 days of osteogenic differentiation, there is no significant difference between the test groups (Figure 3b).

#### 2.1.3. Viability

**Figure 4:**
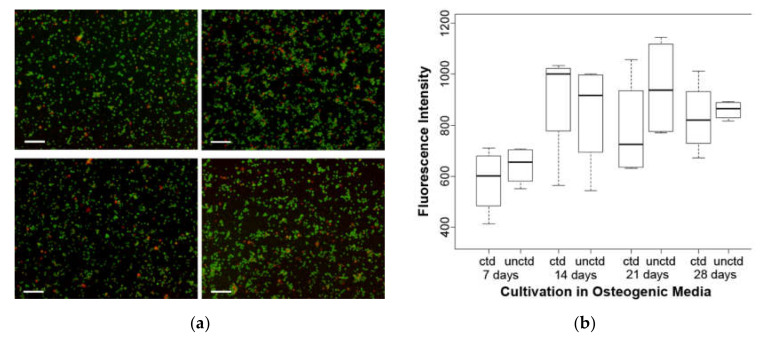
Propidium iodide: (**a**) To visualize the proportion between dead (red) and living (green) cells, cells were photographed after counterstaining them with Calcein-AM, Sigma C1359 (not part of the experiment). Top row: coated, 14 and 28 days. Bottom row: uncoated, 14 and 28 days; bar = 200 µm. (**b**) No statistically significant differences between the two materials are evident. ctd—coated; unctd—uncoated.

The propidium iodide (PI) test shows the viability of the cells in the presence of coated or uncoated grids. PI can only penetrate damaged cells and alters its fluorescence signal, when intercalating between base pairs of DNA and RNA (Figure 4a). The fluorescence intensity, which correspond to the number of living cells, shows no statistically significant differences between coated and uncoated material. This applies to all differentiation times of 7 to 28 days (Figure 4b).

#### 2.1.4. Osteogenic Differentiation

**Figure 5:**
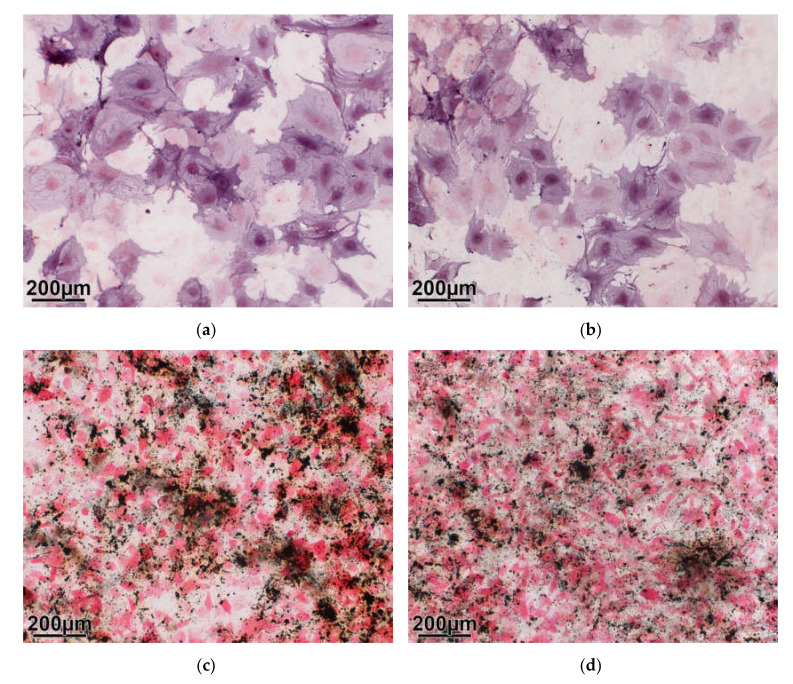
Osteogenic differentiation: (**a**,**b**) The entire cell surfaces show the enzyme reaction with alkaline phosphatase (violet color). (**c**,**d**): The black spots indicate clusters of mineral, which is formed at later stages of osteogenic differentiation (here: 28 days). Coated material (**a**,**c**); uncoated material (**b**,**d**).

As can be shown by the detection of the alkaline phosphatase (Figure 5a,b), a typical marker of osteoblasts, and the formation of mineral, stained with von Kossa (Figure 5c,d), the mesenchymal stroma cells are able to differentiate into osteoblast-like cells in the presence of coated and uncoated material with no qualitative differences between the two test groups.

### 2.2. Small Animal Model

**Figure 6:**
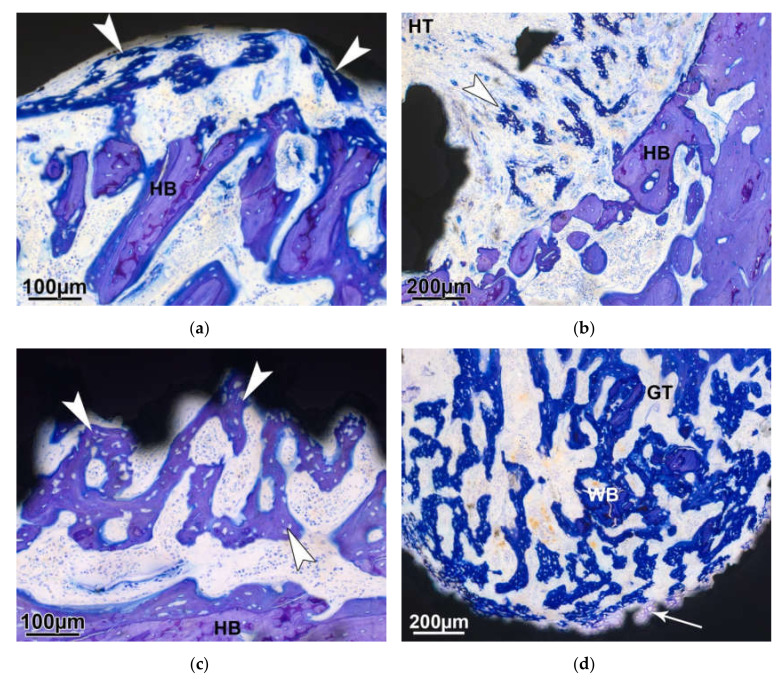
Newly formed woven bone (arrowhead) is visible on the outside of the implant (**a**: 7days and **c**: 14 days). (**b**) At 7 days, woven bone (arrowhead) starts growing through the openings of the implant towards the inside. (**d**) After 14 days, the cavity is largely filled with newly formed trabeculae, which sometimes develop through enchondral ossification (arrow). Coated implant; HB—host bone; WB—woven bone; HT—hematoma residues; and GT—granulation tissue.

Seven days after the surgery, newly formed bone trabeculae develop in the periphery of the defect area around the implant (Figure 6a). The inside of the implant is largely free of bone tissue and shows remnants of hematomas as well as granulation tissue. Starting from the openings of the implant, trabeculae begin to sprout towards the cavity (Figure 6b). Seven days later, trabeculae of woven bone cover the outside (Figure 6c) and fill the cavity inside the implant (Figure 6d), whereas the center remains bone-free. Granulation tissue, characterized by large-lumen blood vessels, is located between the trabeculae (Figure 6d).

**Figure 7:**
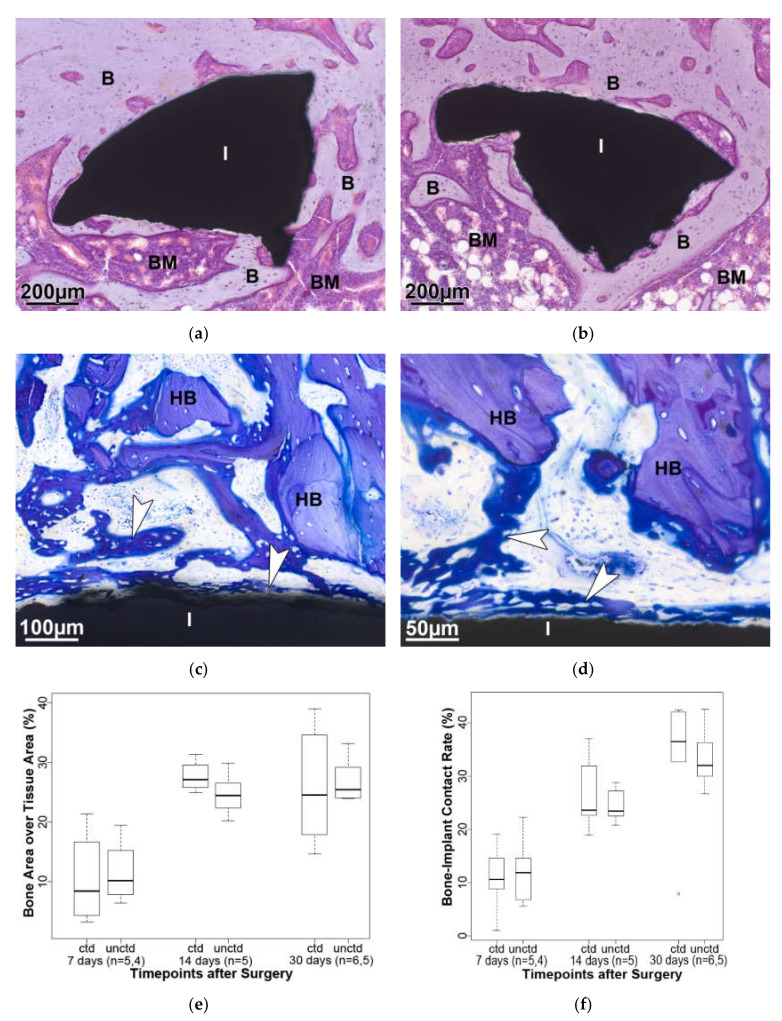
Detailed view of an implant (I) part that is completely consolidated (Hematoxylin-Eosin, 30 days, (**a**) coated, (**b**) uncoated; B—bone and BM—bone marrow). Staining with toluidine blue clearly shows the difference between the host bone (HB) outside the defect zone and the newly formed woven bone (arrowhead) in the interface region between bone and implant; (**c**) coated; (**d**) uncoated, 7 days. (**e**) The bone area over tissue area in the defect area plus 100 µm increase noticeably between 7 and 14 days after surgery and stagnates afterwards. There are no statistically significant differences between coated and uncoated implants. (**f**) During the healing process, an increase in bone-implant contact is visible, but again there is no statistically significant difference between the implant types. ctd—coated; unctd—uncoated.

Finally, 30 days after surgery, part of the granulation tissue is replaced by a bone marrow rich in fat cells (Figure 7a,b). Looking at the amount of bone that has developed in the area of interest, which is the implant area plus 100 µm to cover the implant-bone interface, there is no statistically significant difference between the coated and the uncoated implant at the three different time points (Figure 7e).

Another possibility of quantitatively assessing the amount of newly formed bone is to measure the surface of the implant and to determine the portion that is covered with newly formed bone, the bone-implant contact rate (Figure 7c,d). As expected, the amount of bone increases with the progress of the healing process, but here too no statistically significant difference between the two types of implants were determined (Figure 7f).

**Figure 8:**
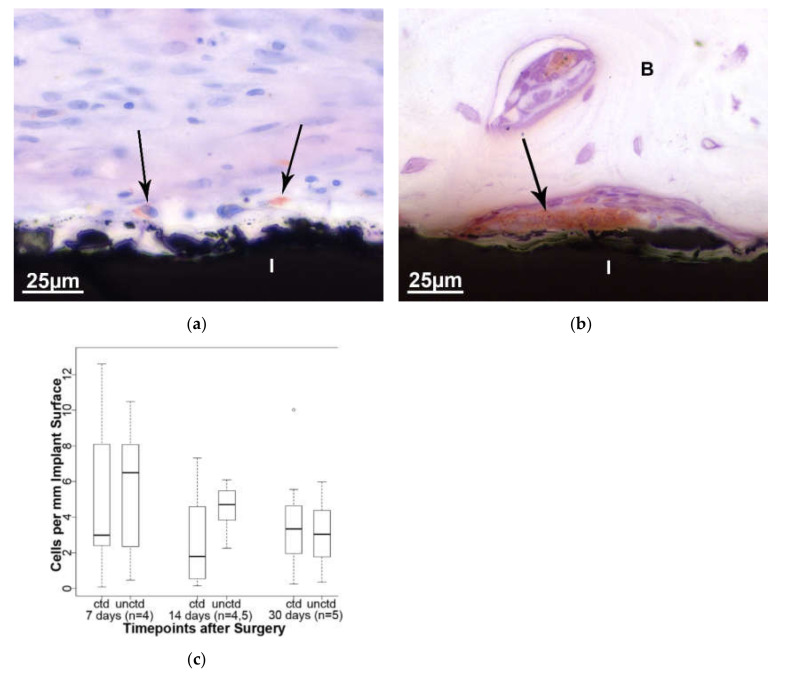
Mononuclear and multinucleated cells stained with ED1 (arrow) are visible on the surface of the implant (I) (**a**, 14 days, coated and **b**, 30 days, uncoated). (**c**) Comparing the number of positive cells among the different time points, no statistically significant difference is evident between the two implant types. ctd—coated and unctd—uncoated.

ED1-positive cells, such as macrophages, osteoclasts, and multinucleated foreign body cells, are located in the defect area and close to the implant (Figure 8a,b). The latter were counted and correlated to the length of the implant and its particles. The number of cells slowly decrease as healing progresses, but again there is no statistically significant difference between coated and uncoated material (Figure 8c).

### 2.3. Large Animal Model

**Figure 9:**
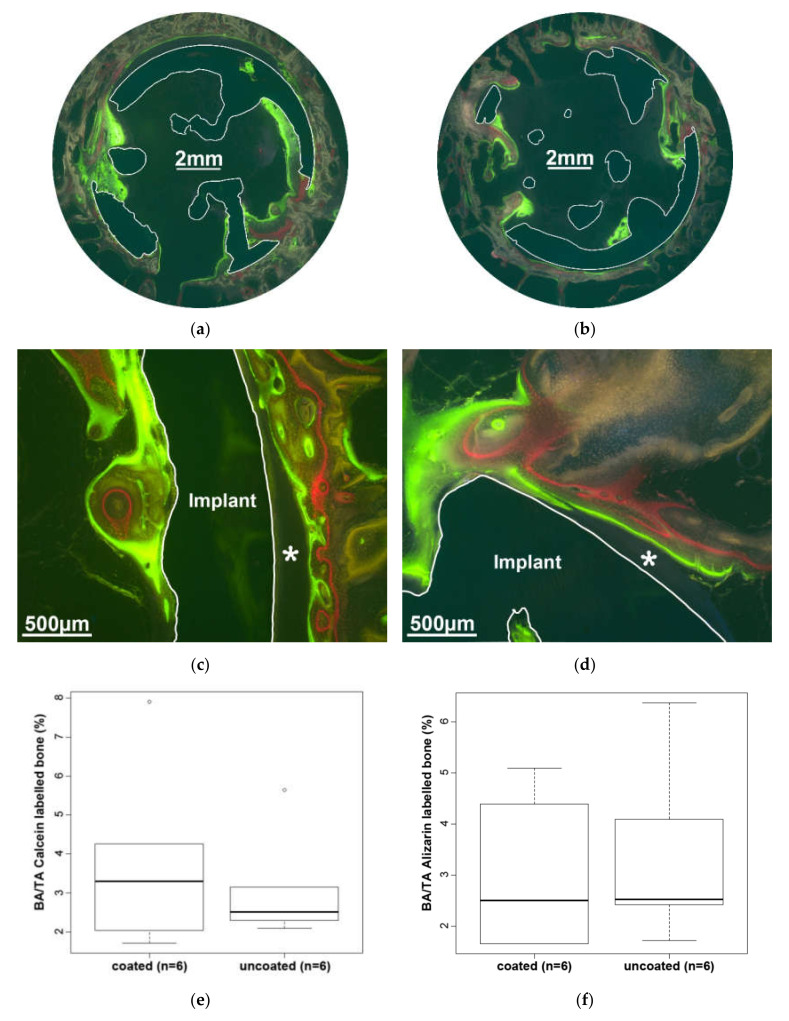
Overview and detail of a coated (**a**,**c**) and uncoated (**b**,**d**) implant (highlighted by a white border). The newly formed bone was labeled by three fluorochromes, Oxytetracycline (yellow), Alizarin Complexone (red), and Calcein (green), and shows the time point at which it was formed. An empty gap (**c**,d*) is visible between the outer surface of the implant and the bone. In the area of interest (AOI), the bone area over tissue area (BA/TA) of both Calcein- (**e**) and Alizarin (**f**)-labeled bone shows no significant differences between coated and uncoated samples. Oxytetracycline-labeled bone was not subject of the investigation.

Newly formed bone spreads through the openings into the cavity and partially covers the inner surface of the implant (Figure 9a,b). Most of the cavity is free of bone and consists of fatty bone marrow. The chronological sequence of the newly formed bone can be clearly seen due to the incorporation of the three fluorochromes.

While the bone usually covers the material on the inside, there is often a microscopic empty gap on the outside of the implant where the bone does not reach the material (Figure 9c,d). This phenomenon occurs with both coated and uncoated implants. In the area of interest (AOI), which is defined by the outline of the implant area plus 400 µm, neither the amount of bone labeled by Alizarin Complexone (red) nor that labeled by Calcein (green), both determined as the bone over tissue area, differs statistically significant between the two test groups (Figure 9e,f).

**Figure 10:**
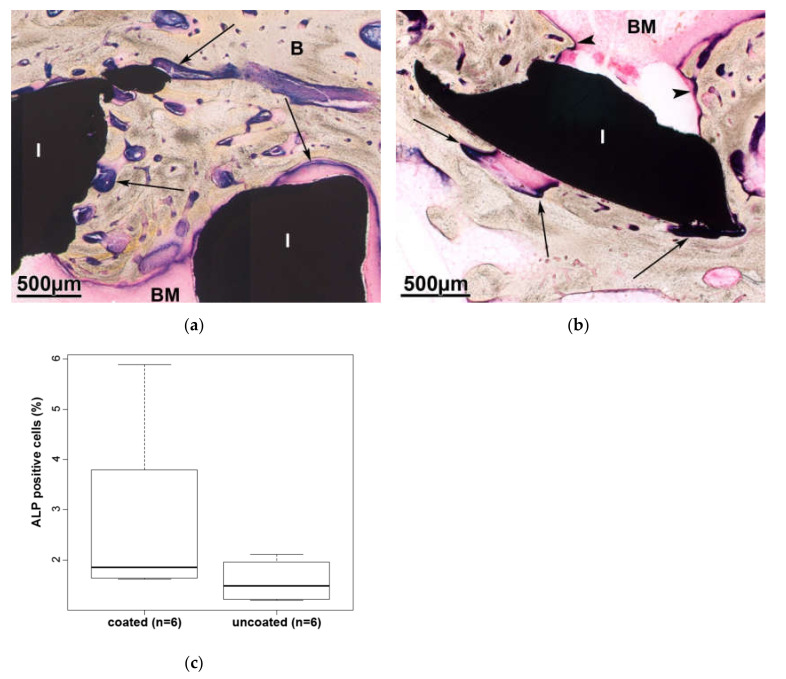
The purple blue coloring indicates the presence of active osteoblasts at the outside (arrow) and in the inside (arrowhead) of the implant (I), coated (**a**) and uncoated (**b**) specimen. (**c**) There are no statistically significant differences in the detection of alkaline phosphatase among the test samples.

The colorimetric proof of alkaline phosphatase activity is a valid way to detect active, bone forming osteoblasts. The purple blue reaction product indicates the presence of osteoblasts. Six months after surgery alkaline phosphatase activity is visibly both outside the implant and lining the bone which is spreading into the implant cavity (Figure 10a,b), which is filled mostly with fatty bone marrow. When measuring the area of the purple blue staining within the AOI and correlating it to the rest of the tissue, there is no statistically significant differences between the test groups (Figure 10c).

**Figure 11:**
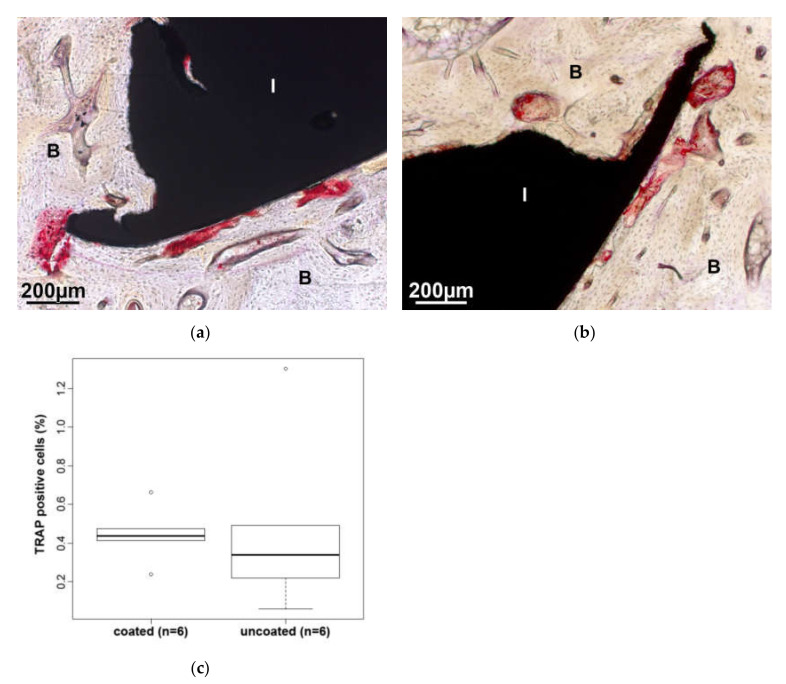
Several red tartrate-resistant acid phosphatase (TRAP)-positive cells are visible lining the coated (**a**) and uncoated (**b**) implant (I). (**c**) The ratio of TRAP-positive cells to bone in the AOI (measured as red areas) shows no significant differences between the two test groups.

Phagocytotic cells can be determined by means of an enzyme reaction, in which the tartrate-resistant acid phosphatase (TRAP) is detected and stained in red. Lining the bone and foreign material, these can be macrophages, osteoclasts and multinucleated foreign body giant cells. The increased accumulation of TRAP-positive cells in the immediate vicinity of the implant (Figure 11a,b) suggests an increased bone metabolism in the interface region and is a sign for an ongoing bone regeneration. Comparing the ratio of TRAP-positive staining to the bone area in and around the two different implants, there are no statistically significant differences (Figure 11c).

## 3. Discussion

The filling of extensive osseous defects presents a major challenge for traumatologists and surgical orthopedists. Because of the aging society, extensive bone defects due to periprosthetic infections or severe bone injuries due to risky sports will lead to an increasing number of necessary replacement operations. For example, in the USA the second most performed tissue transplantation is bone grafting, after blood transfusions [35]. However, the number of donors is limited: the ‘gold standard’, the autogenous bone grafting, usually results in additional surgeries with corresponding complications and risks [4,5,36,37]. Xenogenic transplants are associated with an increased risk of infection. High biocompatibility, safe osteointegration, primary stability, and last but not least availability are required for the use of artificial bone replacement [8]. A material is said to be biocompatible if it does not have any toxic, mutagenic, or carcinogenic effects on the organism [38]. A high degree of osteointegration is required to anchor the implant securely and permanently in the host bone if it is not a resorbable material. Thereby, osteointegration is basically a measure of secondary stability [39,40]. Accordingly, in the past two decades there has been massive research into an ideal bone substitute material with a minimal possible risk for the patient [41]. This resulted in bone replacement materials of non-natural origins such as ceramics, metals, polymers, and cements. Calcium phosphate based materials show an astonishingly high bioactivity [3,7,8], but unlike titanium, they are brittle and do not have a proper mechanical stability and load bearing function [9,10,11], which are essential when used in femoral or tibial bone defects. Frequently described problems when using calcium phosphates are the formation of connective tissue capsules, rejection, and inflammatory reaction, triggered by the hydroxyapatite particles, as well as the formation of seromas and necrosis [42] resulting in a wound healing disorder. Titanium and its alloys are intensively used as a substitute in the human skeletal system, not only because of their primary mechanical stability and corrosion resistance [43], but their biocompatibility [44] and their low tendency to rejection reactions [45], which is primarily due to the formation of a titanium oxide layer (TiO_2_), which develop in the presence of air and forming a boundary between implant and tissue [46,47,48]. Titanium alloys are preferred to commercially pure titanium in loaded situations, such as defects in the long bones, because they provide a higher tensile strength [49,50]. Nowadays, the titanium alloy with niobium is preferably used over vanadium to avoid the risk of toxic vanadium salts [51,52]. Accordingly, we used an implant cast from Ti-6Al-7Nb to test a calcium titanate reaction layer.

Before using an implant material or a special coating in clinical situations, the harmlessness must be ensured. In a first step, the in vitro tests, a possible cytotoxicity and genotoxicity of a foreign material is evaluated, as well as the ability of cells to proliferate and differentiate in the presence of the material [53]. After carrying out various tests, we see that osteoblast-like cells not only colonize the material surface without problems but are also able to proliferate and differentiate. However, since it is not possible to imitate the complexity of bone metabolism in cell culture, we need animal experiments to determine the quality of bone replacement material. In a second step, we therefore tested the implants in a small animal model, the femoral condyle of rats, which is one of the most frequently used animal models for testing bone substitutes [54]. Rats are gentle and easy to handle and show results in a relatively short period of time due to their rapid metabolism. Although the results from a small animal experiment can initially be poorly transferred to humans due to different anatomical and physiological conditions, they allow us to gain an insight into the healing mechanisms and the general biocompatibility of a material. This knowledge allows us to test the implants in a much more complex large animal experiment without fear of serious problems. A sufficient number of studies have shown that the sheep has a weight-bone ratio similar to that of humans. The bone structure as well as the immune, blood and lymphatic systems of sheep are largely homologous to those of humans [55,56,57,58]. This leads to our third step, the test in a critical size defect in sheep femoral condyle.

The grids used in cell culture show a dense colonization of cells, which are able to adhere on the rough surface of the material. As the roughness of an implant increases, the cell population of osteoblasts and thus bone regeneration increases too [59]. In dental surgery, most of the implants have a moderate surface roughness, because several studies see it as an advantage for healing [60,61,62,63,64,65,66]. Our animal experiments show a difference in the way the bone attaches to the outside of the implants. The gaps between the outer surface of the implant and the newly formed bone, which we partially see in the sheep condyles, do not occur in the rat. A reason for this behavior can be the reduced roughness in the sheep implants on the outside, because they were mechanically milled due to geometrical reasons and adherence and migration of bone cells is not possible, when the surface of implants is too smooth [67].

The rat experiments reveal a significant increase in the bone area over tissue area (BA/TA) in the defect area between day 7 and day 14, which stagnates in the further course to day 30. Obviously, a bone remodeling is taking place because BA/TA is stagnating, but the bone-implant contact (BIC) is increasing between 14 and 30 days. In sheep condyles, new bone formation begins in the periphery of the defect and continues through the openings along the inner structure towards the inside of the implant. A large part of the interior, however, is free of bone and filled with fatty bone marrow. Obviously, the bone sees no reason to penetrate further into the implant. This phenomenon epitomizes a problem of metallic implants, the so-called stress shielding, which occurs when the elastic modulus (e-modulus) between the implant and the bone differs significantly. An implant material that is too stiff carries the entire load and leads to bone loss and ultimately implant loosening [68]. Although the e-modulus of titanium and its alloys is lower than that of stainless steel (105 vs. 200 GPa), which is often used as an implant material, it is still significantly higher than in bone (10–40 GPa) [46].

A surface coating or functionalization is carried out to improve the biocompatibility and osseointegration of metallic implants. With titanium and its alloys, the biologically inert surface makes it difficult for bone tissue to bond and therefore increases the risk for loosening the implant. One method to improve the surface properties is to introduce calcium ions to the titanium surface, resulting in the formation of a calcium titanate (CaTiO_3_) layer and thus enhancing the biological activity of the implant [69]. In contrast to a coating with hydroxyapatite, which does not form an intimate connection with the titanium and shows delamination and unpredictable biodegradation [70,71,72], with the consequence of loose implants, the calcium titanate even preserves the surface architecture and roughness of the implant [73]. Several authors describe the benefit of a calcium titanate layer on titanium implants in cell culture experiments [31,74,75,76,77], in femoral condyle [77,78], and tibia [73] of rabbits. Unfortunately, we do not see any significant differences in our experiments between the coated and uncoated implants. As far as animal experiments are concerned, and especially with regard to the sheep experiments, this is possibly due to the small number of animals. In addition, there are clear individual differences between the animals in a group, which leads to a high standard deviation and a lack of statistically significant results. After all, the experiments show a good osseointegration of the implants in rats after 30 days, which is supported by the results of the biomechanical examinations in the same project [79,80]. They show a significant increase in the push-out force compared to the reference (day 0), even if there are no statistically significant differences between the implant types.

Only small amounts of bone were found inside the implant after 6 months in the sheep. Despite the fluorochrome marking, it is not possible to find out whether there was a bone loss inside the implant cavity or if the bone has not grown into the cavity at all. Due to the high elastic modulus of the titanium alloy, both options may be caused by a stress shielding in the months before euthanasia. To clarify the mechanism of new bone formation and possible stress shielding during the healing process of 6 months, we would need more experimental animals, which, however, is complex and expensive in large animal models. Furthermore, it is not possible to predict whether the rat would show bone loss in the defect area after longer time points than 30 days due to stress shielding. When examining the biocompatibility of bone substitute materials, it is essential to include long-term tests also in small animal experiments, especially when using non-removable implant types.

In conclusion, an optimal implant material should offer both initial stability, without the risk of stress shielding, and a functionalization or biomodulation in order to ensure early fixation in the host bone. A promising approach to the latter is the administration of bioactive drugs into the implant material, which are released into the surrounding matrix and induce bone ingrowth and osseointegration. Several methods of drug administration are possible [81]. To quote a few examples, Raina et al. [82] used calcium sulfate/hydroxyapatite inside the hollow core of the implant as a carrier for rhBMP-2 and zoledronic acid. Bai et al. [83] injected a biodegradable supramolecular polysaccharide hydrogel loaded with BMP-2 into the pores of a printed metal scaffold. TiO_2_ nanotube arrays were used to release Metformin in vitro [84] and polyhexamethylene guanidine (PHMG) in vitro and in vivo [85]. In addition to osteoconductive agents, antimicrobial and osteoanabolic drugs can also be delivered locally in this way, thus avoiding the side effects of a systemic administration.

## 4. Materials and Methods

### 4.1. Ethics Permissions

The local ethics commission of the medical faculty of the Justus-Liebig-University, Giessen, Germany approved the use of human cells (05/06 and 106/06) in this experiment.

The local animal care committee approved the use of the rats (Regional Administrative Council Giessen: V54–19 c 20-15 (1) GI 20/14 Nr. 56/2010) and sheep (Regional Administrative Council Darmstadt: V54–19 c 20/15–F31/31) according to §8 Abs. 1 of the German Animal Protection Law.

### 4.2. Material Used in Cell Culture and Animal Models

A casted and coated Ti-6Al-7Nb alloy was used both for cell culture and animal models. The material was casted in Foundry Institute of Rheinisch-Westfälische Technische Hochschule (RWTH) Aachen, Germany [86], then surface cleaned and acid etched in LMW of University of Siegen, Germany [80], and finally coated with a calcium titanate (CaTiO_3_) reaction layer (Federal Institute for Materials Research and Testing (BAM), Research group V4.1 Biomaterials and Implants, Berlin, Germany) [80].

### 4.3. Cell Culture Experiments

**Figure 12:**
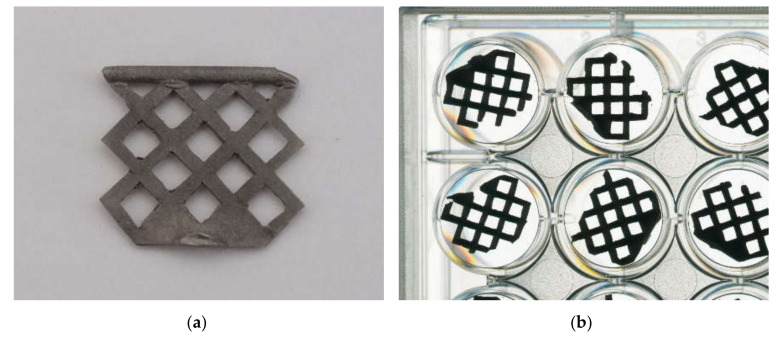
The casted and cleaned grids (**a**) with or without a calcium titanate reaction layer were used in 24-well plates (**b**).

A grid-like structure (Figure 12) with and without a calcium titanate reaction layer was used for cell culture experiments.

Mesenchymal stroma cells derived from human reaming debris [87] of 4 patients were cultivated together with the grids in 24-well plates (20.000 cells/cm^2^) and differentiated for 7, 14, 21, and 28 days in osteogenic media, which contained Dulbecco’s Modified Eagle’s Medium (DMEM) low glucose (PAA, Pasching, Austria, E15-806), 10% fetal calf serum (FKS Gold, PAA, A15-151), 0.1 µM dexamethasone (Sigma-Aldrich, Steinheim, Germany, D8893), 0.05 mM ascorbic acid (Sigma-Aldrich, A4403), and 10 mM glycerol-2-phosphate (Sigma-Aldrich, 50020) at 37 °C and 5% CO_2_. Media was changed every 7 days.

#### 4.3.1. Cell Adherence on the Material

To check the cell adherence on the surface, grids were processed for scanning electron microscopy (SEM) and immunofluorescence (IF). For SEM grids were rinsed in PBS, fixed in 2.5% glutaraldehyde (Roth, Karlsruhe, Germany, 3778) in 0.1 M cacodylate buffer (Roth, 5169), rinsed in 0.1 M cacodylate buffer, dehydrate in a graded series of ethanol (30%, 50%, 70%, 80%, 96%, 100%), immersed first in a mixture (1 + 1) of ethanol and HMDS (Sigma-Aldrich, H4875), then in pure HMDS and air dried. After sputter coating with gold/palladium (Polaron SC7640, Quorum Technologies, Ashford, UK) grids were studied in a LEO 1530 (Zeiss, Oberkochen Germany) with a field emission cathode at 7.5 kV and a backscattered electron (BSE) detector. For IF grids were fixed with methanol 10 min at −20 °C, immersed in a permeabilizing buffer (PBS, 0.3% triton X (Merck, Darmstadt, Germany, 1.08603), 0.1% bovine serum albumin, BSA (Sigma-Aldrich, A8022)), then in a blocking buffer (PBS, 5% BSA) for 30 min., incubated in a monoclonal antibody against cytoskeletal actin (Millipore, Temecula, CA, USA, MAB1501R) 1:100 in blocking buffer for 1 hour, rinsed in PBS, incubated in a biotinylated secondary antibody (Dako, Glostrup, DK, E0354) 1:400 in blocking buffer for 30 min, then in DyLight 488 labelled streptavidin (KPL, Gaithersburg, MD, USA, 042-03-30-00) 1:200 in blocking buffer for 30 min. After mounting with Vectashield Mounting Medium with DAPI (Vector, Burlingame, CA, USA, H-1200) pictures were captured with a triple filter (set25, Zeiss, emission at 460/530/610) on a Zeiss Axiophot2/Axioplan2 and a digital camera DC500 (Leica, Wetzlar, Germany).

#### 4.3.2. Proliferative Capability of Adherent Cells

To test the proliferative capability of the adherent cells the MTT test was used. Again 20,000 cells/cm^2^ were differentiated in an osteogenic media in the presence of the grids in a 24-well plate for 7, 14, 21, and 28 days. 0.5% 3-(4,5-dimethylthiazol-2-yl)-2,5-diphenyltetrazolium bromide (Roth, 4022) were mixed with the existing media and incubated for 4 hours at 37 °C and 5% CO_2_. Sample pictures were taken with an inverted microscope (Leica, Germany, DM IL with a Nikon, Düsseldorf, Germany, camera DS-Fi1). Afterwards cells were lysed in 0.04 n HCl in 2-propanol on a shaker to dissolve the blue formazan crystals produced by the cells. After removing cell residues by centrifugation, the optical density was determined at 570 nm minus 630 nm reference in a WPA Bioware S2100 Spectrophotometer (Biochrom, Cambridge, UK). For each cell line and time point three wells were measured three times and calculated in an excel sheet.

#### 4.3.3. Viability of Cells

To ascertain the viability of the cells in the wells, a propidium iodide / digitonin assay was used. Cells were detached with 0.05% trypsin-EDTA (Gibco, Carlsbad, CA, USA, 15400) and centrifuged together with the original supernatant, in PBS resuspended, again centrifuged, resuspended and mixed with 5 µM propidium iodide (Invitrogen, Carlsbad, CA, USA, P3566) to stain dead cells, distributed in a black 96-well plate and incubated for 20 min at 37 °C. The fluorescent signal was measured in a plate reader (Synergy HT, BioTek, Bad Friedrichshall, Germany) at an excitation of 485/20 and an emission of 645/40. Afterwards every living cell was destroyed with 600 µM digitonin (Serva, Heidelberg, Germany, 19551) and again after an incubation time of 20 min at 37 °C, the propidium iodide signal was measured. To determine the number of living cells, the corrected values from first measurement were subtracted from those of second measurement using Excel (control: PBS plus propidium iodide, then digitonin).

#### 4.3.4. Osteogenic Differentiation

The osteogenic differentiation was qualitatively assessed by visualizing the alkaline phosphatase reaction and the mineral produced by the cells in presence of the grids. Alkaline phosphatase (ALP): after removing the grids, cells were rinsed with PBS, fixed with Methanol, rinsed in double distilled water (ddH_2_O), incubated in 0.1 M Tris buffer (pH 9.4, Trizma Base, Sigma-Aldrich, T6066), incubated with BCIP/NBT (KPL, 50-81-08) for 1 hour at 37 °C, counterstained with nuclear fast red (Roth, N069), covered with Kaiser’s glycerol gelatine (Merck, 1.09242). Mineral was stained with von Kossa: After removing the grids cells were rinsed with PBS, fixed in 4% Paraformaldehyde (Roth, 0335), rinsed in ddH_2_O, incubated in 5% silver nitrate (Sigma-Aldrich, S0139) in ddH_2_O for 60 min, rinsed in water, incubated in a sodium formaldehyde solution (5 g Na_2_Co_3_, Merck, 1.06392; 25 mL 37% formaldehyde solution, Merck 104002; 75 mL water) for 7 min, rinsed in water, reduced in farmer’s reducer (20 mL Sodium thiosulfate (Merck, 106509) 10% in ddH_2_O and 1 mL Formaldehyde 37%) 30 seconds, rinsed in water, counterstained with nuclear fast red and covered with glycerol gelatine. Photos were taken with a Zeiss Axiophot2/Axioplan2 with a digital camera Leica DC500).

### 4.4. Animal Models

**Figure 13:**
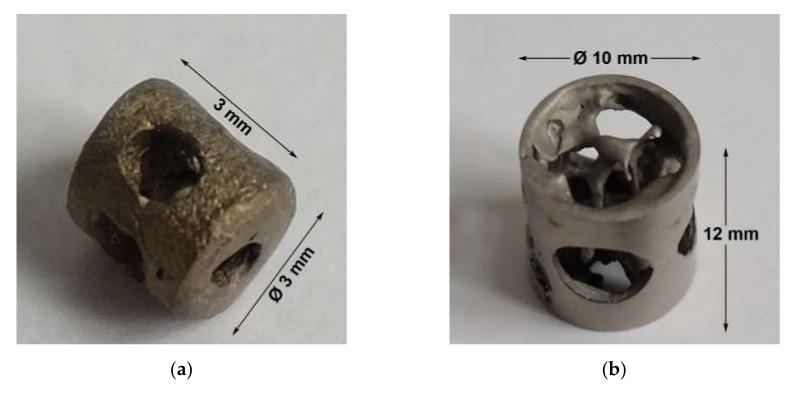
Casted and cleaned implants with and without a calcium titanate reaction layer were used for rats (**a**) and sheep (**b**). The size was 3 mm diameter × 3 mm and 10 mm diameter × 12 mm, respectively. The outer surface of the rat implants shows a macroscopically visible roughness that the sheep implants lack. These were mechanically milled due to geometric reasons.

A cylindrically shaped implant (Figure 13) was used to fill a drill hole defect in rat and sheep femoral condyle, respectively. To obtain a perfectly round cylinder, the sheep implants were mechanically milled before coating.

#### 4.4.1. Small Animal Model (Rat)

32 male Sprague Dawley (Crl:CD(SD)) rats (Charles River, Sulzfeld, Germany) with a mean weight of 463.8 g (389–552 g) were used. All animals were randomly assigned to the time points of 7, 14, and 30 days and the implant with or without reaction layer. Twelve rats were assigned to the 30-day group, in which 1 animal died before surgery. Ten animals were assigned to group 14 and 7 days, respectively. All condyles were embedded in Technovit 9100 (Heraeus Kulzer, Hanau, Germany) for histological examination (Table 1).

**Figure 14:**
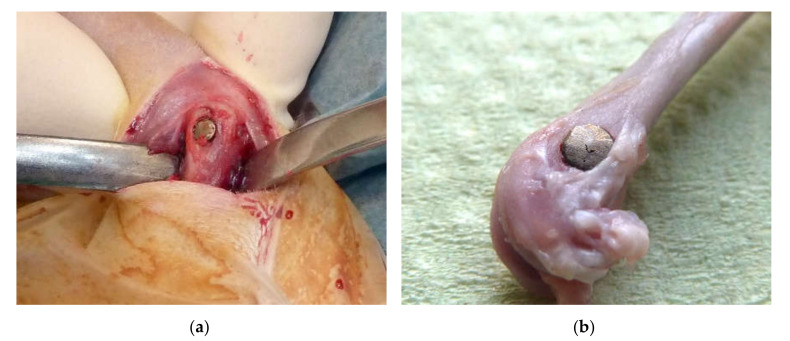
The implant sits press fit inside the defect during the surgery (**a**). An explanted femur shows the position of the implant (**b**).

After anesthesia via injection, rats received a drill hole defect with a spiral drill (Ø 3.2 mm, 3 mm depth) under constant cooling with NaCl in the lateral femoral condyle of the left hindlimb and an implant press fit inside the defect (Figure 14a). Animals were supported with analgesics and euthanatized after 7, 14, and 30 days, respectively.

##### Histology of Rat Femoral Condyles

Femoral condyles were removed (Figure 14b) and fixed in 4% paraformaldehyde and embedded in Technovit 9100 according to manufacturer guidelines.

Condyles were processed sagittally in 3 to 4 grindings with an Exakt (Norderstedt, Germany) cutting/grinding system [88]. Grindings were used for staining with toluidine blue, hematoxylin and eosin (HE), and to detect rat specific CD 68 (ED1). For toluidine blue staining, grindings were etched 45 min with 20% H_2_O_2_, then stained with filtered toluidine blue/pyronin g (8 g di-sodium tetraborate (Merck, 106306), 8 g Toluidine Blue O (Chroma, Münster, Germany, 1B-481) in 800 mL distilled Water (dH_2_O) mixed with 2 g Pyronin G (Merck, 107518) in 200 mL dH_2_O) for 25–30 seconds, dehydrated, covered with Eukitt (Sigma-Aldrich, Germany, 03989) and photographed with a Leica camera DC500 mounted on a Zeiss Axiophot2/Axioplan2. For HE staining, grindings were 3 x 10 min deacrylated in 2-Methoxyethyl acetate (MEA, Merck, 806061), hydrated, stained with filtrated Hematoxylin solution modified acc. to Gill III (Merck, 105174) 40 seconds, blued in tap water, stained with 1% Eosin G (Merck, 115935) for 20 seconds, rinsed, dehydrated, covered with Eukitt and photographed. For immunohistochemistry grindings were deacrylated in 2-Methoxyethyl acetate for 15 min, hydrated in acetone–acetone/washing buffer–washing buffer (TBS-X: Tris-NaCl, pH 7.4: 0,05 M Tris, Roth, 4855; 0,15 M NaCl, Sigma-Aldrich, 31434, 0,025% TritonX-100, Sigma-Aldrich, X100), blocked 10 min in 3% H_2_O_2_ in TBS-X, rinsed in TBS-X, incubated 60 min at room temperature in primary antibody against rat CD68 (ED1, Sigma-Aldrich, MAB 1435) 1:5000 in Antibody Diluent (Dako, S302283), 45 min incubation in secondary antibody (biotinylated rabbit-anti-mouse (Dako, E0354) 1:400 in TBS, 1%BSA (Sigma-Aldrich, A3912), 12.5% Rat Serum (Sigma-Aldrich, R9759), rinsed in TBS-X, incubated in StreptABComplex/HRP (Dako, K0377), after rinsing incubated in NovaRed (Vector, USA, SK-4800), counterstained with Shandon Instant Hematoxylin (Thermo Scientific, Kalamazoo, MI, USA, 6765014), blued in tap water, dehydrated with ethanol and xylene and covered with DePex (Serva, 18243). Pictures were taken with a Leica DC500 mounted on a Zeiss Axiophot2/Axioplan2.

##### Histomorphometry of Rat Femoral Condyles

Using every stained grinding, the whole implant area was photographed with a 10× objective. Pictures were merged with Adobe Photoshop CS5 Extended (San Jose, CA, USA). ED1-positive cells lining the implant surface were counted with the counting tool in Photoshop. The implant surface length was measured with Image Pro Plus 4.5.1 (Media Cybernetics, Rockville, MD, USA). Cells per mm implant length were then calculated in Excel. To determine the amount of bone in the implant area and lining the implant surface, an area of interest was defined, which included the implant area itself and a border of 100 µm around the implant. With the magical wand in Photoshop it was possible to mark and measure the implant and the bone and to calculate the bone area over tissue area (BA/TA) in Excel. Image Pro Plus was used to measure the contact length between the bone and the implant surface (Bone-Implant Contact, BIC) and Excel to calculate the percentage of bone covered implant. The grindings which showed only a small peripheral part of the implant were used for measurement of BIC but not for BA/TA.

#### 4.4.2. Large Animal Model (Sheep)

**Figure 15:**
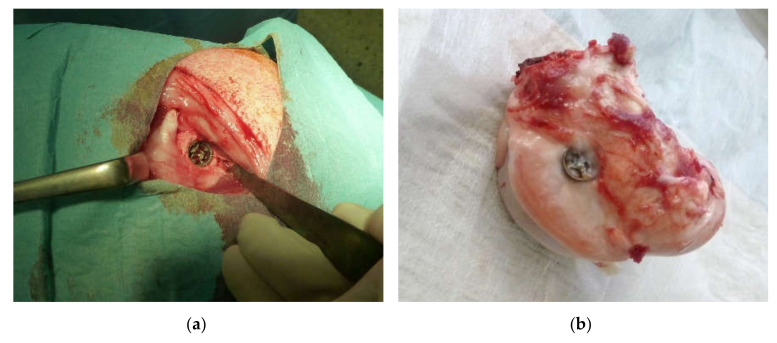
In situ situation during the sheep surgery with the implant inside the drill hole defect (**a**). The distal part of the explanted femur shows the position of the implant (**b**).

Twelve female sheep of the breed Merinolandschaf with a mean weight of 78.6 kg (71–87 kg) and a mean age of 59.5 month (42–78 m) were used in this study. They were randomly assigned to an implant with or without a reaction layer and lived 6 months after surgery. After anesthesia via injection, sheep received a drill hole defect with a Diamond Bone Cutting System (Biomet, Freiburg i. Breisgau, Germany) with a diameter of 11 mm and a depth of 12 mm under constant cooling with NaCl in the medial femoral condyle of the right hindlimb and an implant inside the defect (Figure 15).

After the surgery animals received proper pain medication and left to heal for 6 months. The 12 sheep received sequential fluorochrome injections s.c. to demonstrate bone building at different time points without the need to euthanize additional animals. Oxytetracycline (25 mg/kg, Terramycin LA 20%, Pfizer, New York, NY, USA) was administered on day 29 and 36; Alizarin Complexone (20 mg/kg; Sigma-Aldrich A3882; 3% in Aqua ad inj. + 2% NaHCO_3_ (Roth, HN01) to gain a pH of 7.4) sterile-filtered on day 106 and Calcein (7.5 mg / kg; Sigma-Aldrich C0875; 3% in Aqua ad inj. + 4% NaHCO_3_) sterile-filtered on day 177 and 184.

##### Histology of Sheep Femoral Condyles

The explanted distal part of the femur was cut into 4 or 5 sagittal slices with an Exakt diamond band saw before fixation in 4% Paraformaldehyde and embedment in Technovit 9100. Each embedded slice was processed into 2 to 4 grindings with an Exakt cutting/grinding system. They were stained with Toluidine blue (deacrylated in MEA 2 × 20 min; rehydrated in acetone, acetone/dH_2_O, dH_2_O; stained in Toluidine blue/Pyronin G 10 min; dehydrated shortly in ethanol; covered with Eukitt), processed to reveal the alkaline Phosphatase (deacrylated in MEA; rehydrated in acetone, acetone/dH_2_O, dH_2_O; incubated 10 min in 0.1 M Tris-buffer, pH 9.4; incubated in BCIP/NBT 30 min at 37 °C; counterstained with nuclear fast red; dehydrated in ethanol and xylene and covered with DePex) and tartrate resistant acid phosphatase (deacrylated; rehydrated; incubated in 0.1 M Sodium acetate buffer (Merck 106268) pH 5.2 10 min; stained with substrate (1. dissolve 35 mg Naphthol AS-Tr phosphate disodium salt, Sigma-Aldrich N6125 in 125 µL N,N-Dimethylformamide, Sigma-Aldrich D4551; 2. prepare 25 mL 0.1 M Sodium acetate buffer in a 50 m Falcon tube; 3. dissolve 57.5 mg di-Sodium tartrate dihydrate, Merck 106663 in 1 mL of the buffer; 4. dissolve 35 mg Fast Red TR Salt, Sigma-Aldrich 368881 in 1 mL of the buffer; mix everything together in the remaining buffer) 30 min at 37 °C; counterstained with Shandon Instant Hematoxylin, blued in tap water and covered with glycerol gelatine).

Pictures were taken with a Leica DC500 mounted on a Zeiss Axiophot2/Axioplan2 (toluidine blue, ALP, and TRAP) and a triple fluorescent filter, set25, Zeiss, emission at 460/530/610 (fluorochromes).

##### Histomorphometry of Sheep Femoral Condyles

The whole implant area was photographed with a 2.5× objective. Pictures were merged with Adobe Photoshop CS5 Extended. An area of interest was defined, which included the implant area itself and a border of 400 µm around the implant. With the magical wand in Photoshop it was possible to mark and measure the area of the implant, the bone, TRAP and ALP positive area, and the red (Alizarin) and green (Calcein) fluorescence. The compilation and calculation of the data collected took place in Excel.

### 4.5. Statistical Analysis

For statistical analysis, a one-sample Kolmogorov–Smirnov test and a Shapiro–Wilk normality test were used to determine, if the data show a normal distribution, followed by a two-sample t-test. Calculations and blots were carried out in R, v.3.6.3.

## Figures and Tables

**Table 1:** The table shows how many animals were evaluated.

Time Point	Histology	
	Coated	Uncoated
7 days	5	4 *
14 days	5	5
30 days	6	5 **

* The drill hole in the femoral condyle of one animal was too large, so that the implant could not be inserted. ** One animal died before the surgery.
